# Artificial intelligence literacy in nursing: a concept analysis

**DOI:** 10.3389/fpubh.2026.1874659

**Published:** 2026-06-26

**Authors:** Xu Li, Xu Hu, Huiting Xu, Hailing Ju, Pin Yu

**Affiliations:** 1 Department of Central ICU, The First Affiliated Hospital of Soochow University, Suzhou, China; 2 School of Medicine, Tongji University, Shanghai, China; 3 Department of Operating Room, Qingpu Branch of Zhongshan Hospital Affiliated to Fudan University, Shanghai, China; 4 Department of Nursing, Shanghai Tenth People's Hospital, Shanghai, China

**Keywords:** artificial intelligence, conceptual analysis, literacy, nursing, Rodgers evolutionary conceptual analysis

## Abstract

**Background:**

As artificial intelligence (AI) technologies are increasingly integrated into nursing education and clinical practice, nurses are required to interact with AI systems in complex decision-making contexts. However, the concept of artificial intelligence literacy remains insufficiently defined in nursing, which may limit the development of effective educational strategies and evidence-based practice guidance.

**Methods:**

A comprehensive literature search was conducted in China National Knowledge Infrastructure, Wanfang Data, VIP Database, SinoMed, MEDLINE (via PubMed), Web of Science, and CINAHL (via EBSCOhost) from database inception to October 2025. Studies related to artificial intelligence literacy in nursing were included. Rodgers’ evolutionary concept analysis method was applied to examine the conceptual evolution, defining attributes, antecedents, consequences, related concepts, assessment tools, and exemplar cases of artificial intelligence literacy.

**Results:**

Thirty-four studies were included. Five defining attributes of artificial intelligence literacy in nursing were identified: Theoretical AI Knowledge, Technical Application Proficiency, Critical Evaluation Skills, Awareness of Ethics and Responsibility, and Human-computer collaborative competence. Antecedents were identified at the individual, educational, and organizational levels. Consequences included enhancing the quality of clinical care, strengthening the role of the nursing profession, improving patient experience, enhancing team collaboration, and enhancing educational effectiveness. A conceptual framework of artificial intelligence literacy in nursing was constructed.

**Conclusion:**

Artificial intelligence literacy in nursing extends beyond technical proficiency and represents a context-dependent professional competence grounded in nursing judgment. It is important for supporting safe, ethical, and person-centered nursing care in technology-supported environments.

## 1 Background

Artificial Intelligence (AI) is increasingly being integrated into healthcare systems to support processes such as clinical decision-making, risk warning, personalized medicine, and documentation (1, 2). In nursing practice, AI-driven tools are now being applied in areas such as early warning systems, workload management, patient education, and clinical documentation, reshaping traditional nursing workflows (3–5). Although these technologies are expected to improve the efficiency and quality of healthcare, their rapid diffusion also brings new challenges to healthcare practitioners, especially for nurses who need to interact with AI systems in their daily clinical work (6–8).

Although AI tools are increasingly used in nursing, there remains no unified and clearly defined understanding of the competencies nurses need to use these tools safely, effectively, and ethically. Evidence suggests that overreliance on algorithmic outputs, inadequate understanding of AI mechanisms, and lack of awareness of data biases and system limitations can jeopardize patient safety and the quality of clinical decision-making (9–11). Nurses, as the primary users of bedside AI systems, generally lack systematic training as well as understanding of the mechanisms that generate AI recommendations, and the ability to interpret them with an awareness of their ethical application (12, 13). This cognitive gap raises multiple concerns: overreliance on automated outputs, erosion of critical judgment, and blurred attribution of responsibility in AI-assisted care (14, 15).

Importantly, AI technologies are designed to support, rather than replace, professional nursing judgment (16). International nursing organizations emphasize that nurses remain responsible for interpreting digital information, validating AI outputs, and ensuring that clinical decisions align with patient-specific contexts and ethical standards (17). Consequently, nurses are required not only to operate AI tools but also to critically evaluate their outputs, recognize potential risks, and maintain professional accountability in AI-assisted care environments.

In recent years, the concept of AI literacy has emerged as a framework for describing the knowledge, skills, and critical abilities required to effectively interact with AI systems (18). However, existing research has mainly focused on AI literacy in general education or technical domains, and the theoretical boundaries of AI literacy in nursing are blurred. Existing nursing research has largely focused on knowledge, attitudes, readiness, or intention to use AI, while lacking a theoretically grounded framework to support conceptual operationalization and educational guidance (19). In the absence of clear definitions of AI literacy in the field of nursing, educational initiatives and evaluation tools may focus only on technical skills and neglect core elements such as critical judgment, ethical responsibility, and human-computer collaboration (20). However, these constructs are not conceptually equivalent to AI literacy and cannot fully explain its multidimensional meaning in nursing contexts.

In this study, artificial intelligence literacy in nursing was treated as the core concept of interest. Related terms such as competence, capability, proficiency, and skills were not regarded as interchangeable with AI literacy, but were considered only when they explicitly contributed to the conceptualization, description, or interpretation of AI literacy in nursing contexts.

Therefore, there is a need to clarify the meaning and scope of AI literacy in nursing. This study aimed to analyze the concept of AI literacy in nursing in order to clarify its definition, identify its defining attributes, antecedents, and consequences, distinguish it from related concepts, and construct a nursing-specific conceptual framework.

Rodgers’ evolutionary concept analysis emphasizes the dynamic and context-dependent nature of concepts and is therefore particularly suitable for clarifying emerging constructs in evolving professional contexts (21). In this study, AI refers broadly to both conventional data-driven clinical AI applications and emerging generative AI tools that are increasingly relevant to nursing education and practice. In particular, generative artificial intelligence and large language models introduce additional challenges, including hallucinations, prompt-dependent variability, privacy concerns, and risks related to automated text generation in clinical documentation. Given the rapid development of AI technologies and their expanding role in nursing education, clinical care, and management, AI literacy should not be understood as a static technical ability, but as an evolving concept shaped by professional, ethical, and contextual demands.

Within the nursing context, a concept analysis of AI literacy is needed not only to define the concept itself, but also to clarify its conceptual boundaries and multidimensional structure. Such clarification may help distinguish AI literacy from related concepts, support the development of theoretically grounded assessment tools, and inform curriculum development, clinical training, and policy initiatives related to AI-supported nursing care. Accordingly, this study applied Rodgers’ method to provide both theoretical clarification and practical guidance for the development of AI literacy in nursing.

## 2 Methods

### 2.1 Literature search

A systematic literature search was conducted in China National Knowledge Infrastructure (CNKI), Wanfang Data, VIP Database, SinoMed, MEDLINE (via PubMed), Web of Science and CINAHL (via EBSCOhost). Relevant literature was identified using the search terms “artificial intelligence,” “AI,” “literacy,” “competence,” “competency,” “capability,” “proficiency,” and “skills,” which were combined using Boolean operators as appropriate. Using MEDLINE via PubMed as an example, the search strategy was as follows: ((“Artificial Intelligence”[Mesh]) OR (“artificial intelligence”[Title/Abstract]) OR (“AI”[Title/Abstract])) AND ((“literacy”[Title/Abstract]) OR (“competence”[Title/Abstract]) OR (“competency”[Title/Abstract]) OR (“capability”[Title/Abstract]) OR (“proficiency”[Title/Abstract]) OR (“skills”[Title/Abstract])) AND ((“Nursing”[Mesh]) OR (“Nurses”[Mesh]) OR (“nursing”[Title/Abstract]) OR (“nurse”[Title/Abstract]) OR (“nursing education”[Title/Abstract]) OR (“nursing practice”[Title/Abstract]) OR (“nursing management”[Title/Abstract])). The search covered all records from database inception to October 2025. Because the concept of AI literacy in nursing is still emerging and inconsistently labeled, the search strategy deliberately included adjacent terms such as competence, capability, proficiency, and skills in order to capture literature contributing to conceptual clarification. However, these terms were not assumed to be synonymous with AI literacy and were retained only when they explicitly informed the conceptual meaning of the target construct.

### 2.2 Inclusion and exclusion criteria

#### 2.2.1 Inclusion criteria

Studies were included if they met the following criteria: ① explicitly addressed artificial intelligence literacy or related concepts; ② provided a definition, description, conceptual framework, attribute analysis, or conceptual discussion of artificial intelligence literacy; ③ focused on the nursing field, including nursing education, clinical nursing practice, or nursing management; ④ were available in full text and published in either Chinese or English. Related concepts such as competence, capability, proficiency, and skills were included only when they were explicitly linked to the conceptualization of AI literacy or used to describe its defining features in the nursing context. Studies that merely described the technical use of AI tools, digital ability, or implementation experiences without contributing to conceptual clarification were not treated as evidence for defining AI literacy.

#### 2.2.2 Exclusion criteria

Studies were excluded if they met any of the following criteria: ① focused solely on artificial intelligence technologies or algorithm development without reference to literacy- or competence-related concepts; ② were non–peer-reviewed publications, such as conference abstracts, news reports, or patents, unless a clear conceptual definition was provided; ③ represented duplicate publications or overlapping data; ④ were unavailable in full text; ⑤ were published in languages other than Chinese or English; ⑥ described AI application or adoption in nursing without providing concept-relevant content related to AI literacy. All retrieved records were imported into EndNote 21 for reference management and duplicate removal.

### 2.3 Study selection

**Figure 1:**
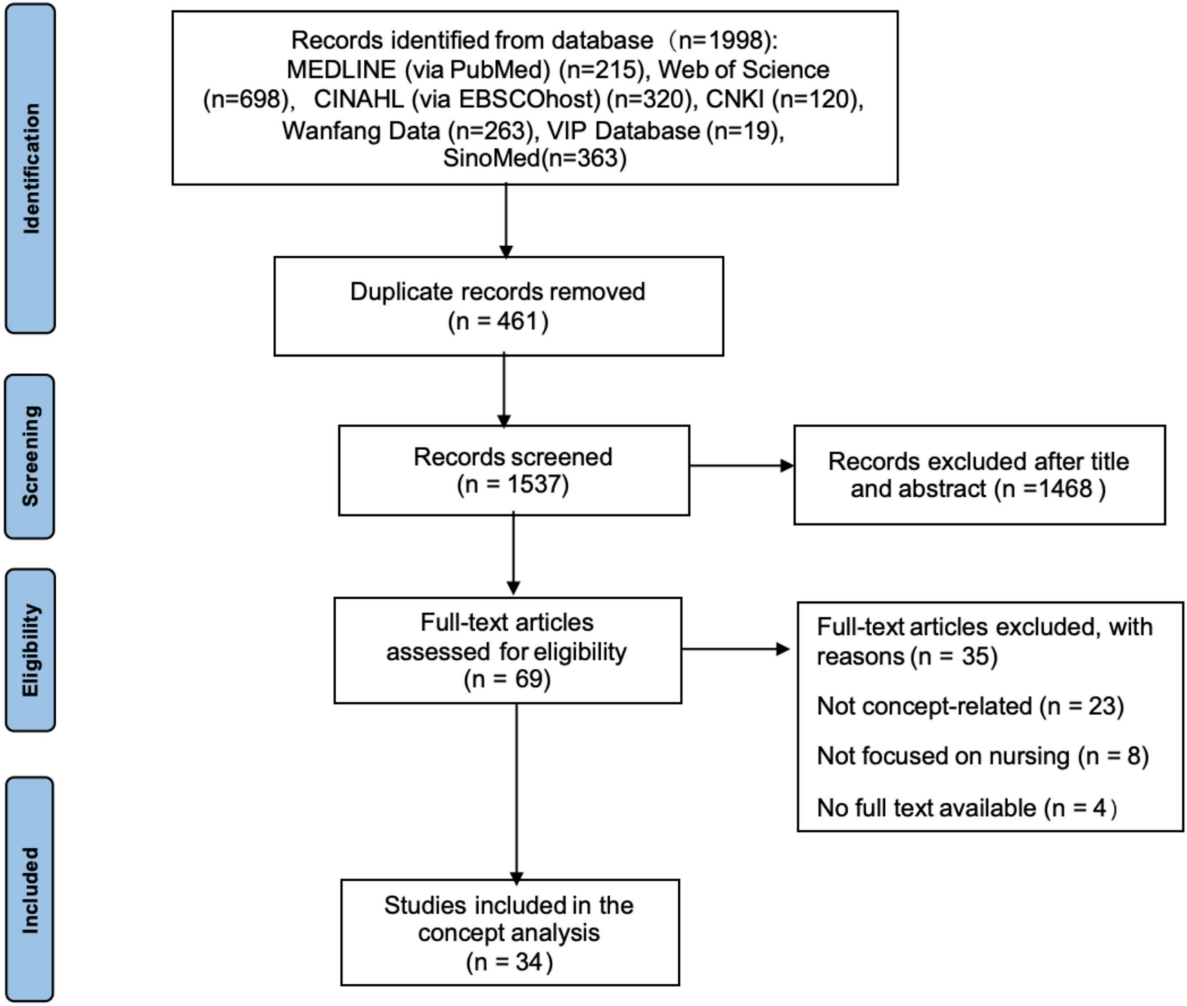
Literature selection process.

Study selection was independently conducted by two researchers. Titles and abstracts were first screened according to the predefined inclusion and exclusion criteria. Full-text articles were then independently reviewed by the same two researchers to determine final eligibility. Any disagreements were resolved through discussion, and when consensus could not be reached, a third senior researcher from the research team was consulted for adjudication. A total of 1,998 records were initially identified. After removal of duplicates, 1,537 records remained. Following title and abstract screening, 69 articles were retained for full-text review. Ultimately, 34 studies met the inclusion criteria and were included in the analysis, comprising 3 Chinese-language and 31 English-language publications. The study selection process is illustrated in Figure 1. The included studies varied in design and purpose, reflecting the evolving and heterogeneous nature of concept-related evidence in this field.

### 2.4 Concept analysis method

Rodgers’ evolutionary concept analysis method (21) was employed to analyze the concept of artificial intelligence literacy in nursing. This approach emphasizes that concepts are dynamic, context-dependent, and continuously shaped by changes in professional and social environments. Consistent with Rodgers’ methodological perspective, the present analysis was conducted as an iterative process involving concept identification, literature selection, examination of conceptual use and development, identification of defining attributes, analysis of antecedents and consequences, differentiation from related concepts, review of assessment approaches, and generation of implications for future conceptual development. These analytic components were used in this study as an operational structure to organize the concept analysis, rather than as a rigid sequence of fixed phases.

To enhance methodological transparency, a standardized data extraction matrix was developed based on Rodgers’ framework. Extracted information included publication characteristics, concept-related definitions or descriptions, defining attributes, antecedents, consequences, related concepts, assessment tools, and exemplar cases. The included literature comprised diverse source types, including conceptual papers, surveys, qualitative studies, mixed-methods studies, scale development studies, review papers, and discussion or framework-oriented articles, consistent with Rodgers’ emphasis on capturing the contextual and evolving use of concepts. The full data extraction matrix for all included studies is provided in Supplementary Table 1.

Two researchers independently extracted and compared concept-related data from the included studies. Through iterative reading, constant comparison, and team discussion, preliminary categories were developed and gradually refined into higher-order conceptual themes. Formal intercoder reliability statistics were not calculated because the analysis aimed at interpretive conceptual synthesis rather than quantitative content coding. Instead, credibility was supported through independent extraction, constant comparison, and team discussion.

## 3 Results

### 3.1 The origin and evolution of the concept of AI literacy

The concept of AI literacy can be traced back to early exploratory discussions in the 1970s. Early scholars, such as Agre et al. (22), began to consider the basic abilities required for engaging with artificial intelligence from the perspective of AI practitioners. However, because AI technology was still in its infancy at that time, these early discussions did not attract widespread attention. As a result, AI-related abilities were more often subsumed under broader concepts such as information literacy and digital literacy, and AI literacy had not yet emerged as an independent concept.

With the gradual expansion of AI applications, scholars began to question whether the public and learners needed a distinct form of literacy to understand and engage with AI. Yoko (23) broadened the discussion beyond technical mastery, arguing that AI literacy should also involve ways of thinking and the capacity to respond to future technological change. Kandlhofer et al. (24) further emphasized that AI literacy should be understood and discussed as an independent concept rather than merely an extension of other forms of literacy.

A major step in the formalization of the concept occurred in 2019, when UNESCO explicitly highlighted AI literacy within an international policy framework, describing core AI literacy as involving an understanding of how AI collects, cleans, manipulates, and analyses data, and how algorithms identify patterns and relationships in such data (25). Long and Magerko (18) provided one of the most influential and systematic definitions of AI literacy, describing it as a set of knowledge, skills, and attitudes that enable individuals to understand the fundamentals of AI, critically evaluate and use AI systems, and interact effectively with them. They also proposed 15 core competencies, which laid an important foundation for subsequent research.

From 2020 onward, AI literacy entered a phase of rapid expansion and conceptual deepening. Scholars increasingly linked AI literacy to broader issues such as social impact, technological bias, and ethical responsibility. For example, Ng et al. (26) synthesized existing definitions and proposed that AI literacy encompasses perceptions, competencies, and qualities that support the effective and ethical use of AI, including four dimensions: understanding, use, evaluation, and ethics. Hermann (27) further emphasized the social and ethical implications of AI, connecting AI literacy with data literacy, critical thinking, and awareness of bias. Carolus et al. (28) later argued that AI literacy is reflected in the capacity to engage with AI in an autonomous, rational, and critical manner in everyday life.

More recently, AI literacy has moved beyond the domain of general digital education and has become increasingly contextualized within professional practice. In engineering, for example, AI literacy places greater emphasis on understanding models, prompt design, and evaluating output quality (29). In organizational and management contexts, it has evolved from a basic tool-use ability to a hierarchical competence that shapes AI orientation and implementation across different organizational levels (30). In healthcare, AI literacy has become even more context-dependent. Healthcare professionals are expected not only to understand and use AI tools, but also to interpret algorithmic outputs, recognize bias and risk, and make appropriate judgments within clinical contexts. In nursing in particular, these demands are closely tied to professional accountability, patient safety, and the ability to verify and contextualize AI recommendations through nursing expertise (31). Therefore, nursing has become one of the most important professional contexts in which the concept of AI literacy is being refined and operationalized. Although the included literature showed broad convergence in recognizing knowledge, application, evaluation, and ethics as key components of artificial intelligence literacy, some contextual differences in emphasis were observed. Studies from Western contexts tended to place relatively greater emphasis on autonomy, critical evaluation, and ethical governance, whereas studies from Eastern contexts more often highlighted educational preparedness, implementation conditions, and organizational support. These differences do not indicate fundamentally different concepts, but they suggest that the relative emphasis of artificial intelligence literacy may vary across professional and cultural settings.

### 3.2 Defining attributes of AI literacy in the nursing domain

The inductive analysis of the included literature in this study reveals that AI literacy in the nursing domain presents a multidimensional structural feature, and its core attributes can be summarized into five dimensions: Theoretical AI Knowledge, Technical Application Proficiency, Critical Evaluation Skills, Awareness of Ethics and Responsibility, and Human-computer collaborative competence, which together form the basis of the caregiver’s competence to make decisions and practice in intelligent nursing contexts.

#### 3.2.1 Theoretical AI knowledge

Theoretical AI Knowledge is the foundational dimension of AI literacy, referring to nurses’ understanding of the working mechanisms, algorithmic logic, applicable conditions, and potential risks of AI. In nursing contexts, if it remains only at the surface level of use, it will directly limit the nursing staff’s ability to interpret and make clinical judgments about AI-generated recommendations. Tseng et al.’s (32) study further found that some nursing students were able to skillfully invoke the AI system, but showed significant difficulty when asked to explain the basis of AI recommendations, suggesting that there is a disconnect between knowledge acquisition and practical application. Boztepe et al. (33) suggests that nursing AI literacy should follow a progressive structure of “understanding-assessment-application,” with knowledge being a prerequisite for operational and assessment skills. Theoretical AI Knowledge is therefore characterized by being able to explain the “why” of AI rather than just being able to see and accept the results.

#### 3.2.2 Technical application proficiency

Technical Application Proficiency refers to the ability of nursing staff to use artificial intelligence systems safely and effectively across clinical care, health education, documentation, and decision-support activities, and to apply these skills appropriately in different nursing contexts. Ng et al. (26) regarded technical application proficiency as an operational manifestation of AI literacy that extends from digital literacy, and it is the core of transforming technical understanding into practical operation. Dodson et al. (34) found that, in the absence of systematic training, nursing students were often able to perform AI operations, but were unable to integrate the tool output with clinical reasoning and individual patient differences, resulting in a “can-use-but-cannot-judge” approach. Si (35) demonstrated that structured guidance (including hands-on exercises, case studies, and reflective discussions) can significantly improve skills and clinical transfer through a staged teaching intervention. Thus, Technical Application Proficiency is a demonstration of the ability to move from understanding to practice, as evidenced by: being able to use, being able to use correctly, and being able to use in clinical situations.

#### 3.2.3 Critical evaluation skills

Critical evaluation is one of the core attributes of AI literacy, which refers to the ability of caregivers to rejudge and proofread AI outputs rather than relying on them mechanically. Simms (36) emphasizes that the focus of AI education is not on teaching system functionality, but on developing the ability to recognize “black box bias” and “model assumptions.” In the absence of critical assessment, the convenience of AI translates into an over-reliance on the system, which can undermine professional judgment. Sengul et al.’s (37) study showed that caregivers tended to follow recommendations rather than interpret them when using clinical decision-support AI, suggesting that a lack of critical assessment skills poses a risk of executive dependency. Chen et al. (38) pointed out that the evolutionary trend of AI literacy is changing from “being able to use” to “being able to explain, question, and rejudge”; Jiang et al. (39) further clarified that the essence of AI literacy is a kind of technological critical ability. In the context of generative AI, critical evaluation also involves recognizing hallucinated content, prompt-dependent variability, and documentation-related inaccuracies that may arise from large language models. For nurses, this means that outputs generated by artificial intelligence should not be accepted at face value, but must be verified against clinical evidence, patient context, and professional judgment. Therefore, critical evaluation is reflected in not blindly accepting or rejecting artificial intelligence suggestions, but in being able to explain why they should or should not be acted upon in specific nursing situations.

#### 3.2.4 Awareness of ethics and responsibility

Awareness of ethics and responsibility refers to the ability of nursing staff to clarify decision-making authority and responsibility when using AI, to protect patients’ rights and interests, and to maintain professional subjectivity. Agaoglu et al. (40) found that nurses generally have a general sense of uncertainty about “who bears the ultimate responsibility” when an AI is involved in clinical decision-making, so it is important that this be part of AI literacy. Therefore, a sense of responsibility must be part of AI literacy. Song et al. (41) suggested that nursing staff need to be able to negotiate human and AI decisions to ensure that nursing judgment is not replaced by the system. The American Nurses Association (ANA) (16) further emphasized in its position statement on the use of AI that AI can assist, but not replace, professional nursing judgment and that nurses should be held accountable for the outcome. Therefore, the sense of ethics and responsibility is reflected in the fact that the AI may participate in decision-making, but the final judgment and responsibility belong to the nurse.

#### 3.2.5 Human-computer collaborative competence

Human-computer collaborative competence is an advanced and comprehensive manifestation of AI literacy and refers to the ability of nursing staff to integrate AI information within the clinical team and to work with AI on nursing analysis, decision-making, and communication rather than viewing AI as a one-way tool. He et al. (42) noted that nursing practice in the AI era is shifting from executive to human-computer collaborative work. El Arab et al. (5) further noted that nurses should screen, filter, and interpret AI recommendations in the clinic before communicating them to the team or patient, reflecting their role as mediators. Thus, human-computer collaboration is reflected in making decisions with the AI rather than being replaced by the AI.

### 3.3 Antecedents of AI literacy in nursing

The formation of AI literacy is not the result of the action of a single factor, but is contributed to by a combination of influences at the individual, educational, and organizational levels. In this analysis, antecedents refer to conditions that appear to facilitate the emergence, development, or enactment of AI literacy in nursing. The competency base at the individual level determines whether caregivers have the prerequisites to learn AI, the instructional design at the educational level influences whether AI literacy can be effectively constructed and transferred to the clinic, and the system and culture at the organizational level determine whether AI literacy can be implemented and sustained in real-life nursing scenarios.

#### 3.3.1 Individual level

Individual-level antecedents are the basis for the construction of AI literacy among caregivers, which refers to the competency base of digital literacy, critical thinking skills, clinical experience, and professional identity that caregivers themselves possess. Studies have shown that caregivers with higher levels of digital literacy can master the operation and information access of AI systems more smoothly, thereby reducing technical barriers to use (32). Critical thinking skills are also particularly important in AI contexts, helping caregivers to rejudge AI-generated conclusions rather than adopting them blindly (19), and those with more clinical experience are more likely to recognize possible inconsistencies between AI outputs and the patient’s real condition, thus making a more robust decision between “reference” and “adoption” (40). Professional identity also affects the role of caregivers in AI-assisted situations, with those with stronger identities more likely to maintain subjectivity in care decision-making rather than delegating judgmental responsibility to the AI system (31).

#### 3.3.2 Educational level

The educational level antecedent is a key facilitator of AI literacy formation and refers to the ability of AI literacy to migrate into nursing practice through curricula, teaching, faculty, and learning materials. It has been noted that intellectual explanations of AI concepts and tools alone cannot truly enhance the AI literacy of nursing staff and must be integrated with clinical contexts in order to develop competence (43). It is contextualized and problem-oriented teaching that can help learners understand AI and leverage its value in real nursing scenarios, facilitating the transfer of competencies to practice (35). Interdisciplinary educational models can also enhance caregivers’ understanding of AI models, algorithmic logic, and sources of risk, enabling them to interpret and assess the reliability of AI outputs (34). Faculty AI competency can also directly impact learning outcomes; if instructors lack experience with AI applications or critical perspectives, the depth of AI literacy developed by students will be limited (44).

#### 3.3.3 Organizational level

Organizational level antecedents that determine whether AI literacy can be truly implemented in the clinic on the ground are whether the healthcare organization has intelligent system resources, operational protocols, training systems, and a patient safety culture. If the healthcare organization does not provide a stable and accessible AI system, lacks standardized operating procedures, or lacks training and support mechanisms, it is difficult to apply AI literacy in practice, even if the caregivers have it (45). Whether a healthcare organization has a patient safety culture also influences caregivers’ attitudes toward the adoption of AI-assisted recommendations, and if the organization promotes the use of and requires the retention of the clinical verification process, caregivers may maintain a more subjective approach and professional judgment in AI collaboration (46). Thus, organizational-level factors determine whether AI literacy can be translated from competence to implementable motivation.

### 3.4 Consequences of AI literacy in nursing

The included literature suggests that AI literacy in nursing may have implications for multiple domains of practice and professional development, including the quality of clinical care, the role of the nursing profession, patient experience, team collaboration, and educational effectiveness. However, some of these consequences—particularly those related to patient safety and clinical outcomes—remain conceptually inferred and are not yet supported by extensive empirical evidence.

#### 3.4.1 Enhancing the quality of clinical care

Higher levels of AI literacy may enable nursing staff to interpret and use AI-generated information more appropriately in assessment, monitoring, and intervention. In high-alert scenarios, nurses are able to make informed clinical judgments and interventions based on data provided by AI to reduce the occurrence of adverse events and facilitate the implementation of individualized care (47). For example, in scenarios such as fall risk prediction, pressure ulcer early warning and vital sign trend analysis, caregivers can rely on AI’s early recognition signals to take care measures in advance, which not only helps reduce the incidence of adverse events in patients, but also further promotes precision and individualized care, and comprehensively improves patient safety and overall quality of care. However, the direct relationship between AI literacy and patient safety outcomes has not yet been sufficiently established through empirical studies and therefore should be interpreted with caution.

#### 3.4.2 Strengthening the role of the nursing profession

In the clinical context of AI-assisted decision-making, if nursing staff lack sufficient AI literacy, it is often easy to over-rely on the technology and allow “AI to replace nursing judgment,” thus weakening the subjectivity of the nursing profession in clinical decision-making (40). On the contrary, caregivers with good AI literacy can clearly delineate the boundary between “AI provides reference suggestions” and “caregivers make the final decision,” and maintain their responsibility for clinical judgment and outcomes while improving decision-making efficiency through AI. The professional status of the subject is maintained while the clinical judgment and outcome are maintained through the use of AI to improve the efficiency of decision-making (31). This clarification of role boundaries not only helps to maintain the professional autonomy of the nursing discipline but also provides a solid foundation for the continued development and value of nursing in an intelligent healthcare system.

#### 3.4.3 Improving patient experience

Caregivers with good AI literacy can clearly explain to patients the sources, roles, and limitations of AI suggestions, thereby effectively alleviating patients’ concerns about the possible risks of intelligent systems and enhancing their understanding of and trust in the care process (35). In contexts such as health education, behavioral guidance, and treatment adherence support, caregivers can also make full use of the visualization and interpretable output of the AI system to help patients understand health information more intuitively and promote their active participation in shared decision-making, a process that not only enhances the patient’s experience of the hospital, but also further improves overall satisfaction with the care (44).

#### 3.4.4 Enhancing team collaboration

Nursing staff with higher AI literacy can communicate more effectively with physicians, information technology teams, and administrators about problems and improvement needs in AI applications, thus facilitating the optimization and integration of intelligent systems in clinical pathways (34). On this basis, the improvement of AI literacy not only strengthens the ability to apply technology at the individual level but also promotes the extension of human-computer collaboration from individual practice to the team and system level. Through this interdisciplinary collaboration model, nursing staff can play a leading role with the support of AI, promoting the formation of a new model of intelligent nursing care that is “AI-enabled, multidisciplinary collaboration, and nursing-led decision-making” (43).

#### 3.4.5 Enhancing educational effectiveness

By incorporating AI literacy into the teaching objectives and evaluation indexes of the nursing education system, nursing education can realize the transformation of teaching from the traditional teaching mode of “focusing on the transmission of knowledge” to the teaching oriented on “constructing competence in real situations.” The transformation of teaching. For students, the AI literacy framework not only helps them understand the basic principles and operation mechanisms of AI, but also guides them to evaluate the logic and reliability of AI outputs during the learning process and make appropriate clinical judgments in specific nursing contexts. Through this ability transfer, students can gradually develop from “being able to use AI” to “using AI well,” thus promoting the overall improvement of clinical competence (48). Meanwhile, for teachers, having AI literacy will help promote the reform of course content and teaching methods, and promote the application of innovative teaching strategies such as interdisciplinary teaching, contextualized case study, and reflective learning, so that nursing education can better meet the new trend of intelligent nursing development (49).

### 3.5 Conceptual framework of AI literacy in nursing

**Figure 2:**
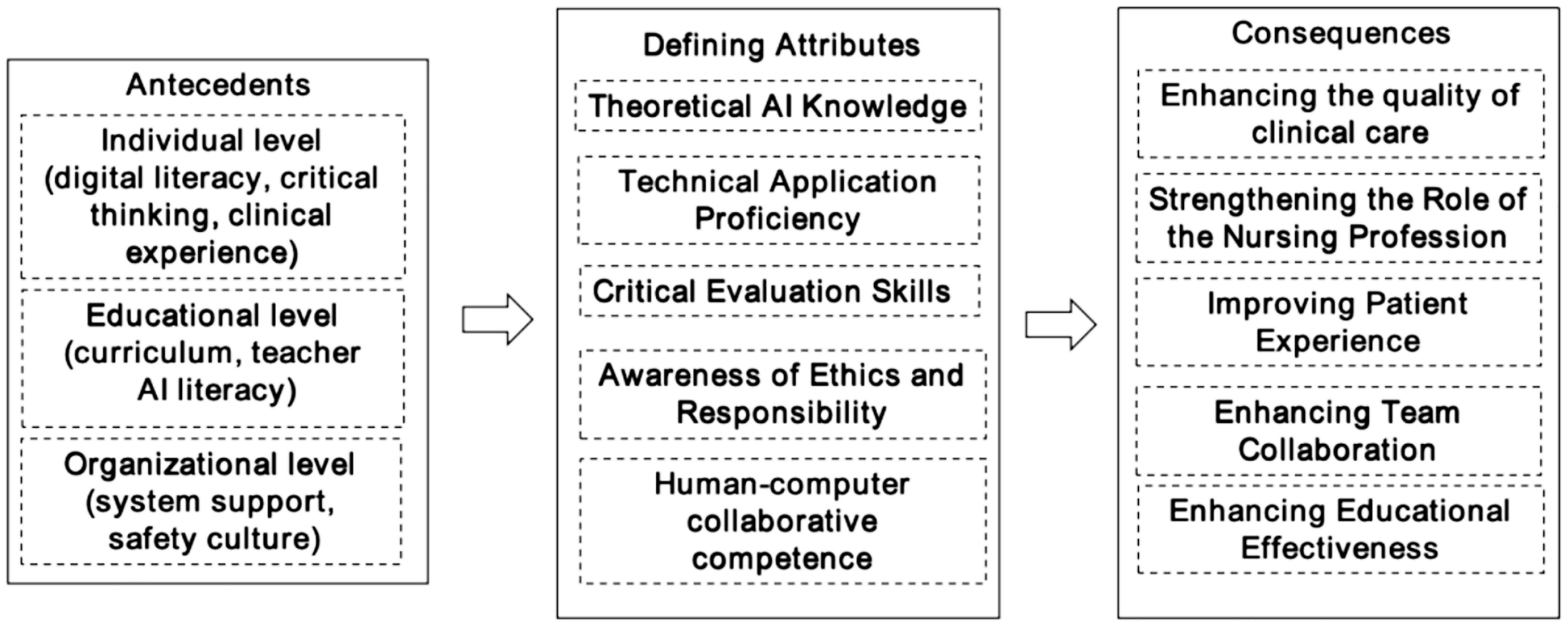
Conceptual framework for AI literacy in nursing.

Based on the present analysis, artificial intelligence literacy in nursing may be understood as a context-dependent professional competence that includes: understanding how AI systems work and where their limitations lie; applying AI tools appropriately in nursing tasks; critically appraising AI-generated outputs; maintaining ethical and professional accountability in AI-assisted care; and collaborating effectively with AI while retaining nursing judgment and patient-centered responsibility. Figure 2 illustrates how antecedents at the individual, educational, and organizational levels jointly shape the defining attributes of artificial intelligence literacy in nursing, which in turn contribute to the identified consequence domains.

### 3.6 Identification of AI literacy concepts

AI literacy intersects with concepts such as digital literacy and information literacy in the field of nursing and health education, but each has a different focus and connotative boundaries, and conceptual identification is needed to clarify the core concepts analyzed in this study.

#### 3.6.1 Difference between AI literacy and digital literacy

Digital literacy is the combined ability of individuals to effectively, critically, creatively, and responsibly use digital technologies in digital environments for information acquisition, communication and collaboration, content creation, problem solving, and social engagement (50), whereas AI literacy requires not only the mastery of digital skills, but emphasizes an understanding of the logic of how AI models work, the sources of data, the risks of bias, and the boundaries of application understanding, and critical assessment and judgmental oversight in clinical decision making.

#### 3.6.2 Differences between AI literacy and information literacy

Information literacy is concerned with the ability to utilize information systems for data recording, transfer, retrieval, and clinical information management, with a focus on information processing and information system use (51), whereas AI literacy is concerned with how to interpret and evaluate AI-generated results and how to maintain professional subjectivity and ethical responsibility in decision making.

### 3.7 Assessment tools for AI literacy

Existing tools for assessing AI literacy are mainly general instruments rather than nursing-specific measures. Among the tools identified in the included literature, the Artificial Intelligence Literacy Scale and the Meta AI Literacy Scale are the most frequently reported. However, no nursing-specific validated instrument for assessing AI literacy was identified, which further highlights the need for future scale development in this field. Although these tools provide useful foundations for assessing general AI literacy, they do not fully capture several dimensions highlighted in this analysis, particularly Critical Evaluation Skills, Awareness of Ethics and Responsibility, and Human-computer collaborative competence in nursing care. This gap underscores the urgency of developing a nursing context-specific AI literacy instrument. The conceptual framework developed in this study may therefore serve as a useful basis for defining the core content domains of future nursing-focused AI literacy measures.

#### 3.7.1 Artificial intelligence literacy scale, AILS

The Artificial Intelligence Literacy Scale (AILS) was developed by Wang et al. in 2023 to assess general AI literacy across four dimensions: awareness, use, assessment, and ethics (52). The scale includes 12 items scored on a 7-point Likert scale, with higher total scores indicating higher levels of AI literacy. Reported psychometric properties showed good reliability and validity, with a Cronbach’s alpha of 0.92 and a content validity index ranging from 0.80 to 0.85.

#### 3.7.2 Meta AI literacy scale, MAILS

The Meta AI Literacy Scale (MAILS) was developed by Carolus et al. in 2023 to provide a broader assessment of AI literacy (53). It includes 34 items across seven dimensions: use and apply AI, understand AI, detect AI, AI ethics, create AI, AI self-efficacy, and AI self-competence. Items are rated on a 11-point Likert scale ranging from 0 to 10, with higher scores indicating higher levels of AI literacy. The scale demonstrated good internal consistency, with an overall Cronbach’s alpha of 0.93 and subscale alphas ranging from 0.81 to 0.88. Factor analytic testing also supported the adequacy of the scale structure.

### 3.8 Typical cases

By analyzing the concepts of typical cases, the connotation and practical significance of AI literacy can be more intuitively understood and identified. Take a seriously ill patient as an example: the patient was prompted as “high risk” by the intelligent pressure ulcer risk assessment system due to being bedridden for a long period of time. In the face of this warning, some caregivers directly followed the standardized care path provided by the system to implement routine measures such as turning, decompression, etc., without further evaluating the patient’s skin integrity, nutritional status, and positional tolerance, resulting in a more mechanical care plan and a lack of individualized care. On the other hand, nursing staff with higher AI literacy demonstrated more comprehensive judgment; they would first understand and explain the source and basis of the systematic warning, and then reassess the patient’s actual signs and medical history to adjust interventions promptly to make nursing care more precise, and at the same time, they would also explain the necessity of the nursing interventions to the patient to promote the patient’s understanding and participation. This case fully demonstrates the critical role of AI literacy in understanding AI output, conducting critical assessment, and human-computer collaboration.

## 4 Discussion

### 4.1 Conceptual contribution

This concept analysis clarifies artificial intelligence literacy as a nursing-specific, multidimensional, and context-dependent concept rather than a purely technical skill or a general attitude toward technology. Existing studies in nursing have often focused on knowledge, attitudes, readiness, or intention to use artificial intelligence, while paying less attention to the conceptual structure of artificial intelligence literacy itself. By identifying its defining attributes, antecedents, consequences, related concepts, and a conceptual framework, the present study offers a clearer account of what artificial intelligence literacy means in nursing and why it should be distinguished from adjacent constructs.

A key contribution of this study is that it situates artificial intelligence literacy within the professional logic of nursing. The findings suggest that artificial intelligence literacy in nursing is not limited to understanding or operating artificial intelligence tools. Rather, it involves the ability to interpret outputs critically, recognize ethical and professional responsibilities, and collaborate with artificial intelligence while retaining nursing judgment. In this sense, the concept is more appropriately understood as a professional competence shaped by clinical context, patient safety, and the values of nursing practice.

### 4.2 Comparison with existing frameworks

The framework identified in this study is broadly consistent with existing artificial intelligence literacy models, particularly those proposed by Long and Magerko (18) and by Ng et al. (20), which conceptualize artificial intelligence literacy as extending beyond basic technical knowledge to include understanding, use, evaluation, interaction, and ethics. Subsequent work has further emphasized critical engagement, ethical awareness, and context-sensitive interaction with artificial intelligence (27, 28). Similar competency-oriented discussions have also emerged across other health disciplines. For example, recent reviews in health professional education have highlighted artificial intelligence fundamentals, ethical and legal considerations, evaluation of outputs, communication, and teamwork as key competency domains (54, 55), while research in medical education has shown that artificial intelligence literacy is increasingly treated as a measurable and teachable construct rather than merely a matter of attitude or acceptance (56).

However, the present concept analysis suggests that artificial intelligence literacy in nursing has a more profession-specific meaning than these general frameworks imply. In nursing contexts, artificial intelligence is embedded in care processes that require context-sensitive interpretation, professional accountability, and responsibility for patient outcomes (16, 17, 31). For this reason, the present framework places greater emphasis on Critical Evaluation Skills, Awareness of Ethics and Responsibility, and Human-computer collaborative competence, which appear particularly salient in nursing practice. This nursing-specific emphasis is also consistent with recent health care literature showing that clinicians need competencies not only in using artificial intelligence tools, but also in verifying outputs, recognizing limitations, and integrating them responsibly into practice (12, 54).

Taken together, these comparisons suggest that general artificial intelligence literacy frameworks provide an important conceptual foundation, but they require contextual refinement when applied to nursing. The present analysis therefore does not replace earlier models; rather, it extends them by showing that, in nursing, artificial intelligence literacy is closely tied to professional judgment, patient-centered responsibility, and the capacity to work with artificial intelligence without relinquishing nursing subjectivity.

### 4.3 Implications for future research

As artificial intelligence continues to expand across nursing education and practice, several priorities for future research remain. First, studies should further identify which dimensions of artificial intelligence literacy are most critical in nursing, particularly ethical reasoning, critical evaluation, practical application, and human-computer collaborative competence. Second, empirical research is needed to examine how artificial intelligence literacy may influence patient safety through mechanisms such as verification behaviors, error detection, risk awareness, and documentation review. Third, educational research should move beyond general recommendations and specify appropriate instructional approaches, target learner groups, and measurable outcomes. Finally, there is a clear and urgent need to develop and validate nursing-specific assessment instruments for artificial intelligence literacy. The conceptual framework proposed in this study may provide the core content domains for such instruments, particularly Theoretical AI Knowledge, Technical Application Proficiency, Critical Evaluation Skills, Awareness of Ethics and Responsibility, and Human-computer collaborative competence. This may support the future development of a nursing context-specific AI literacy scale.

### 4.4 Limitations

This study has several limitations. First, as a concept analysis based on published literature, the findings were shaped by the scope, quality, and reporting of existing studies, and may not fully capture unpublished or practice-based understandings of AI literacy in nursing. Second, only Chinese- and English-language literature was included, which may have introduced language bias and limited the representation of conceptual developments in other linguistic or cultural contexts. Third, although Rodgers’ evolutionary concept analysis is well suited to examining dynamic and context-dependent concepts, the analytic process inevitably involved interpretive judgment. While data extraction and category refinement were conducted through researcher discussion and consensus, formal intercoder reliability statistics were not calculated, which may have increased the risk of subjective interpretation. Finally, because AI technologies and their nursing applications are evolving rapidly, the meaning and boundaries of AI literacy may continue to change over time. Accordingly, the conceptual framework proposed in this study should be considered provisional and open to future refinement as the field develops.

## 5 Conclusion

This study clarifies artificial intelligence literacy in nursing as a multidimensional and context-dependent competence that extends beyond technical proficiency to include Theoretical AI Knowledge, Technical Application Proficiency, Critical Evaluation Skills, Awareness of Ethics and Responsibility, and Human-computer collaborative competence. Compared with previous general frameworks, it provides a clearer nursing-specific account of the concept’s attributes, antecedents, consequences, and conceptual boundaries. By constructing a conceptual framework for artificial intelligence literacy in nursing, this study helps reduce ambiguity in the existing literature and provides a conceptual basis for future education, practice, and measurement development. Ultimately, the goal of enhancing nursing AI literacy is not only to support the effective use of artificial intelligence, but also to uphold and strengthen the humanistic care values of the nursing profession in the era of human-computer collaboration.
